# Temporal visuomotor synchrony induces embodiment towards an avatar with biomechanically impossible arm movements

**DOI:** 10.1177/20416695231211699

**Published:** 2023-11-07

**Authors:** Harin Hapuarachchi, Hiroki Ishimoto, Michiteru Kitazaki, Maki Sugimoto, Masahiko Inami

**Affiliations:** Department of Computer Science and Engineering, 13129 Toyohashi University of Technology, Toyohashi, Japan; Department of Information and Computer Science, Faculty of Science and Technology, Keio University, Yokohama, Japan; Research Center for Advanced Science and Technology, University of Tokyo, Tokyo, Japan

**Keywords:** unnatural avatar, sense of agency, sense of ownership, sense of embodiment, visuomotor synchrony

## Abstract

Visuomotor synchrony in time and space induces a sense of embodiment towards virtual bodies experienced in first-person view using Virtual Reality (VR). Here, we investigated whether temporal visuomotor synchrony affects avatar embodiment even when the movements of the virtual arms are spatially altered from those of the user in a non-human-like manner. In a within-subjects design VR experiment, participants performed a reaching task controlling an avatar whose lower arms bent in inversed and biomechanically impossible directions from the elbow joints. They performed the reaching task using this “unnatural avatar” as well as a “natural avatar,” whose arm movements and positions spatially matched the user. The reaching tasks were performed with and without a one second delay between the real and virtual movements. While the senses of body ownership and agency towards the unnatural avatar were significantly lower compared to those towards the natural avatar, temporal visuomotor synchrony did significantly increase the sense of embodiment towards the unnatural avatar as well as the natural avatar. These results suggest that temporal visuomotor synchrony is crucial for inducing embodiment even when the spatial match between the real and virtual limbs is disrupted with movements outside the pre-existing cognitive representations of the human body.

All our lives, we have been experiencing ourselves being immersed inside a body that can be moved as we intend, to interact with our surroundings. Various sensations we continuously receive through vision, touch, and proprioception during such interactions allow us to distinguish what is “ours” among the objects we perceive. The term “body ownership” has been given to this self-attribution of the body (Gallagher, 2000) that presumably depends on the afferent sensations arising within the body itself (Tsakiris et al., 2007). Our sense of body ownership also depends on the coherence of the current sensory input with the pre-existing cognitive representations of the body (Tsakiris & Haggard, 2005) making it difficult for us to feel embodied in new objects or bodies apart from our own.

However, manipulating visual, tactile, proprioceptive, and motor sensations in specific ways can trick our minds into believing external or virtual objects to be parts of us. One classic body ownership illusion found in literature is the rubber hand illusion (RHI) (Botvinick & Cohen, 1998; Farne et al., 2000; Rorden et al., 1999), where participants reported a feeling of owning a rubber hand after observing it being stroked with a paint brush while the hidden real hand was also synchronously stroked. Immediately after experiencing the RHI, with their eyes closed, participants typically tend to point at a position drifted away from their own hand and towards the rubber hand when asked to point at their real hand, showing a proprioceptive drift (Botvinick & Cohen, 1998; Costantini & Haggard, 2007; Tsakiris & Haggard, 2005; Tosi et al., 2023). Some studies also report an increase in skin conductance levels when the rubber hand is threatened during the experience of the illusion (Armel & Ramachandran, 2003; Honma et al., 2009; Tsuji et al., 2013). However, when the stroking on the real hand is asynchronous/incongruent with that on the rubber hand, the illusion is known to occur to a much lesser extent (Armel & Ramachandran, 2003) meaning that the illusion is mainly caused by the multisensory integration of what is seen (the visual input of the rubber hand being stroked) and what is felt (the synchronous tactile sensation on the real hand).

In addition to such visuotactile correlations, synchronous visuoproprioceptive (passive as well as active movements) and visuomotor (active movements) correlations are also known to induce illusions of owning surrogate body parts (Dummer et al., 2009; Kalckert & Ehrsson, 2012; Kalckert & Ehrsson, 2014; Kondo et al., 2020b; Tsakiris et al., 2006; Walsh et al., 2011). When it comes to synchronous visuomotor stimulations, immersive Virtual Reality (VR) with real-time body tracking has been used to investigate further into the underlying mechanisms of illusory body ownership (Gonzalez-Franco et al., 2010; Inoue & Kitazaki, 2021; Petkova & Ehrsson, 2008; Sanchez-Vives et al., 2010; Slater et al., 2010; Weijs et al., 2021). Some studies even suggest that synchronous visuomotor stimulations induce stronger body ownership illusions compared to synchronous visuotactile stimulations (Kokkinara & Slater, 2014), highlighting the importance of visuomotor synchrony for embodiment.

However, most previous embodiment studies have investigated the temporal effect of visuomotor synchrony on the sense of embodiment while being limited to the physical constraints of the human body and maintaining the human-like nature of the virtual movements (Gonzalez-Franco et al., 2010; Sanchez-Vives et al., 2010; Weijs et al., 2021). Although temporally synchronous visuomotor stimulations are known to induce a sense of embodiment even towards various types of bodies, such as bodies with different skin colors (Peck et al., 2013), sizes (Banakou et al., 2013), and invisible bodies (Kondo et al., 2018; Kondo et al., 2020a), the movements of the avatars and participants have been spatially congruent in those studies too, where virtual limb positions and orientations spatially coincide with real limb positions and orientations in synchronous conditions. Here, we were interested in understanding how manipulating the spatial aspects of visuomotor synchrony affects the sense of embodiment.

A previous study on spatially scrambled body parts has shown that spatial alteration of limb positions disrupts full body illusions while maintaining ownership for the scrambled (individual) body parts (Kondo et al., 2020a). However, in the scrambled body experiment by Kondo et al., while the hands and feet were presented in scrambled locations in a virtual environment, the motion directions of the virtual hands and feet were the same as those of the participant (Kondo et al., 2020a). Here, we were interested in understanding how embodiment is affected when movements are altered instead of the positions of the individual limbs. Would temporal visuomotor synchrony induce a sense of embodiment even towards bodies with such biomechanically impossible body movements? And how would temporal synchrony in the absence of spatial synchrony compare to spatial synchrony in the absence of temporal synchrony when it comes to sense of embodiment? To find answers to these questions, in a within-subjects design experiment, we had 24 participants perform a reaching task in a virtual environment controlling an avatar that bent the lower arms from the elbow joints in the direction opposite to their real lower arms (see Figure 1).

**Figure 1.**
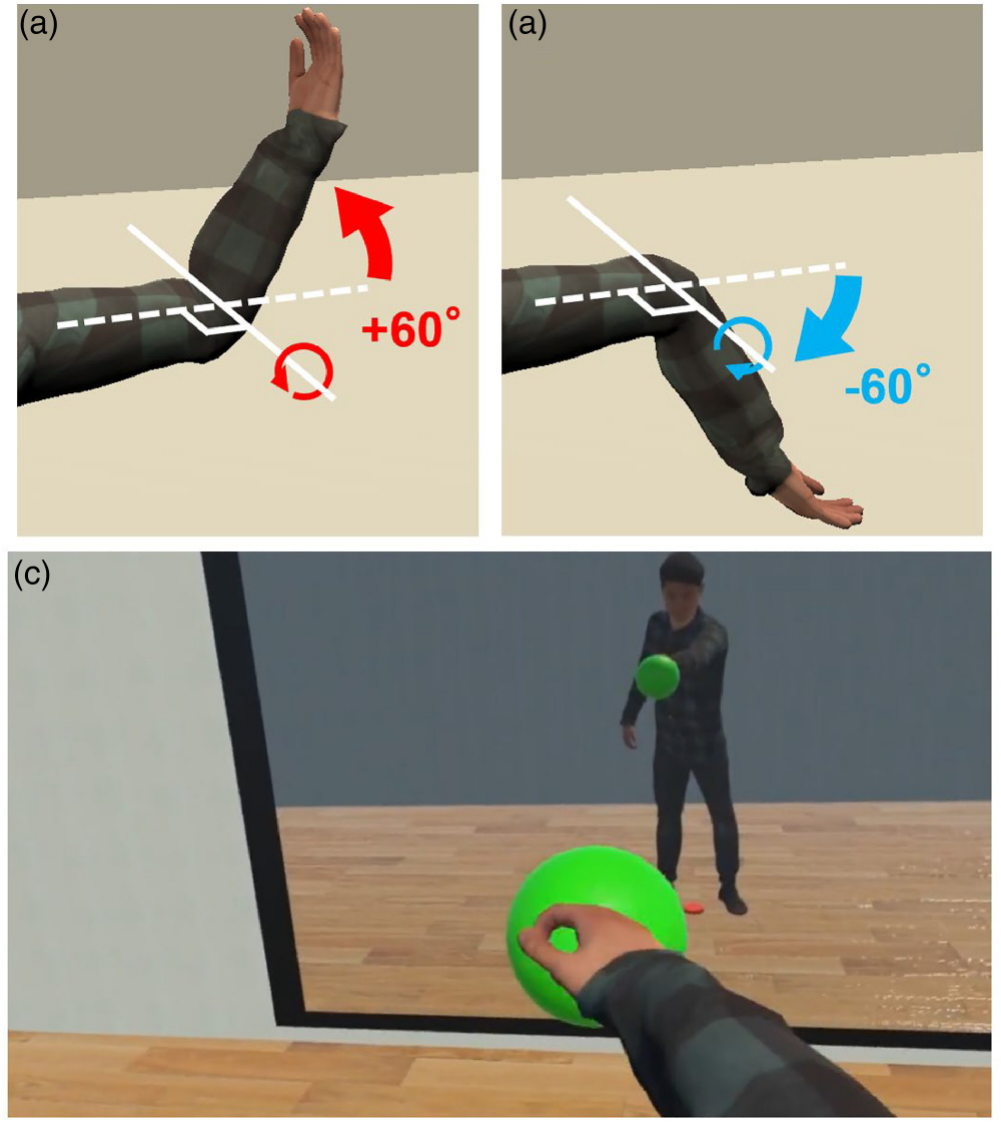
The two types of avatars used in the experiment and the experiment task: (a) *Natural avatar:* Lower arms of the avatar bent in the same direction as the participant, (b) *Unnatural avatar:* Lower arms of the avatar bent in the opposite direction of the participant's movement. (c) *Experiment task:* Participants controlled natural and unnatural avatars in first-person perspective in a virtual environment to perform a reaching task.

We investigated how temporal visuomotor synchrony affects embodiment of such an “unnatural avatar” compared to a “natural avatar” that moved with the unaltered movements of the participant. The experiment consisted of two synchronicity conditions, the “synchronous” condition, where the timing of the virtual movements matched the timing of the real movements, and the “asynchronous” condition, where the virtual movements were delayed for 1 s from the real movements. Participants performed the reaching task in both synchronous and asynchronous conditions using the unnatural avatar as well as the natural avatar, and we measured their sense of embodiment, and skin conductance in response to a visual stimulus of a knife threatening the lower arms of the avatar at the end of each session (see Figure 2). Sense of embodiment is known to consist of three subcomponents (Kilteni et al., 2012): the sense of self-location, which is the ability to perceive the location of one's body parts (Blanke & Metzinger, 2009; Lenggenhager et al., 2009), the sense of agency, which is the sense of having control of motion (Blanke & Metzinger, 2009; Haggard, 2005), and the sense of body ownership, which refers to one's self-attribution of the body (Gallagher, 2000). Therefore, to get a broader idea of sense of embodiment, in this study, we measured the sense of agency as well as the sense of body ownership of the participants using questionnaire items based on the avatar embodiment questionnaire by Gonzalez-Franco and Peck (Gonzalez-Franco & Peck, 2018).

**Figure 2.**
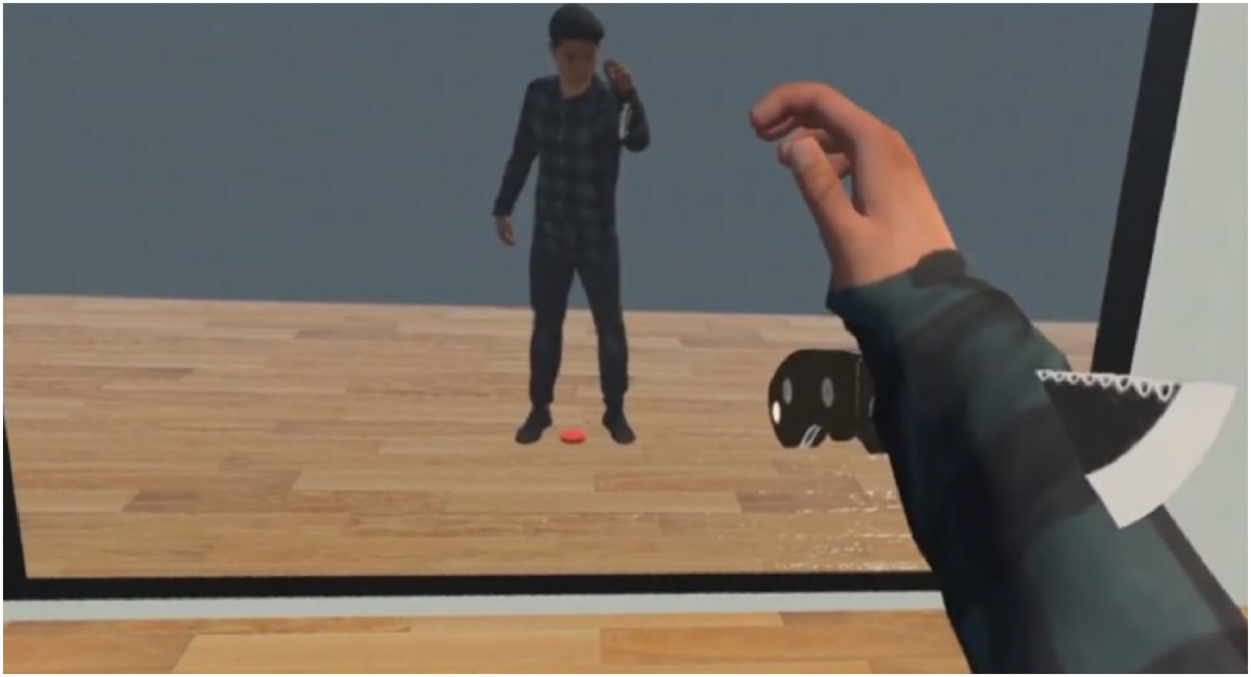
Stimulus of the knife at the end of the reaching task. Example scene of the avatar's lower arm being stabbed by a knife at the end of the natural avatar condition.

Studies on visuomotor adaptation suggest that our brain is highly flexible in integrating visual and motor information to maintain accurate motor outputs (Held & Hein, 1958; Krakauer, 2009; Redding & Wallace, 1993; Redding et al., 2005) and visual stability (Becklen et al., 1984; Kitazaki, 2013; Stratton, 1896; Welch, 1969; Wallach, 1987). When the visual feedback of a movement mismatches the proprioceptive feedback, the brain often recalibrates to minimize the discrepancy, leading to a shift in the perceived body ownership towards the visual representation (Synofzik et al., 2006; Synofzik et al., 2008). Studies on virtual co-embodiment also have shown that individuals prioritize the observed movements of their virtual avatar over their own movements even when the actual movement of participant deviates from the avatar's movement (Hagiwara et al., 2020). If the brain prioritizes such visual information over proprioceptive feedback during visuomotor mismatches, such adaptability may also have implications on our perception of body ownership and sense of agency with biomechanically impossible movements. Therefore, we predicted that participants will still adapt to the arms bending in opposite directions and feel a sense of embodiment towards the unnatural avatar in our study, as long as the visual movements temporally match their body movements. Based on our sensitivity to temporal discrepancies in multisensory integration (Berger et al., 2023), we predicted that temporal visuomotor synchrony will play a more dominant role than spatial synchrony in inducing sense of embodiment. In other words, even if the movements are spatially incongruent, as long as they are temporally synchronous, we predicted that the participants will experience a sense of embodiment stronger than what they feel with natural movements with a temporal delay. We also hypothesized there to be a significant increase in both sense of agency and sense of body ownership towards the unnatural avatar (as well as towards the natural avatar) in the synchronous condition compared to the asynchronous condition. However due to the mismatches between proprioception and visual feedback while using the unnatural avatar, we hypothesized the sense agency and body ownership scores towards the unnatural avatar to be significantly lower than those towards the natural avatar.

Changes in Skin Conductance in response to threatening stimuli to rubber hands (Armel & Ramachandran, 2003; Honma et al., 2009; Tsuji et al., 2013) as well as virtual avatars (Hapuarachchi & Kitazaki, 2022; Petkova & Ehrsson, 2008; Petkova et al., 2011;Yuan & Steed, 2010) have been measured in embodiment related studies as a physiological measure of body ownership. Following such work, as a measure of sense of body ownership, we measured the physiological response to a threat to the virtual lower arms using skin conductance response (SCR), expecting a higher SCR at experiment conditions that induced a higher sense of body ownership towards the avatar (Figure 2).

## Methods

### Participants

Twenty-four university students (all males, mean age 21.2, SD 1.04) participated in the experiment. The sample size was determined by a power analysis with repeated measures ANOVA (4 within factors: 2 × 2 conditions), an effect size (*f*) of 0.25 (medium), an alpha of 0.05, and power of 0.80, and an expected correlation of 0.5 between repeated measures using G*Power 3.1 (Faul et al., 2007; Faul et al., 2009). All participants had normal or corrected-to-normal eyesight, and the study procedures were designed to minimize disclosure of the experimental hypothesis (participants were not told what the hypotheses or the objectives of the study were). They provided informed consent before the experiment. The methods of the experiment were approved by the Ethical Committee for Human-Subject Research at the Toyohashi University of Technology, and all experiments were carried out in accordance with the relevant guidelines and regulations.

### Setup and Apparatus

The movements of each participant were measured by a motion capture system (OptiTrack FLEX 3, 100 Hz). The virtual environment and the avatar were displayed through a head-mounted display (HMD: HTC VIVE Pro Eye, 1440 × 1600 pixels per eye, 90 × 110deg, 90 Hz refresh). The virtual environment and experiment task were created using Unity (2017.3.0f3). Two vibration motors that vibrated at 217 Hz were attached to the two palms of the participants that vibrated each time the corresponding virtual hand of the avatar reached the targets. BIOPAC MP 160 and wireless PPG and EDA amplifiers (BN-PPGED) were used to record the SCR data. A wireless transmitter (BN-PPGED) was placed on each participant's wrist. Two disposable electrodes (EL507) were pasted on the distal phalanges of their middle and ring fingers. Two lead cables (BN-EDALEAD2) connected the wireless transmitter to the electrodes. The SCR data were recorded at 1000 Hz.

### Stimuli and Conditions

**Figure 3.**
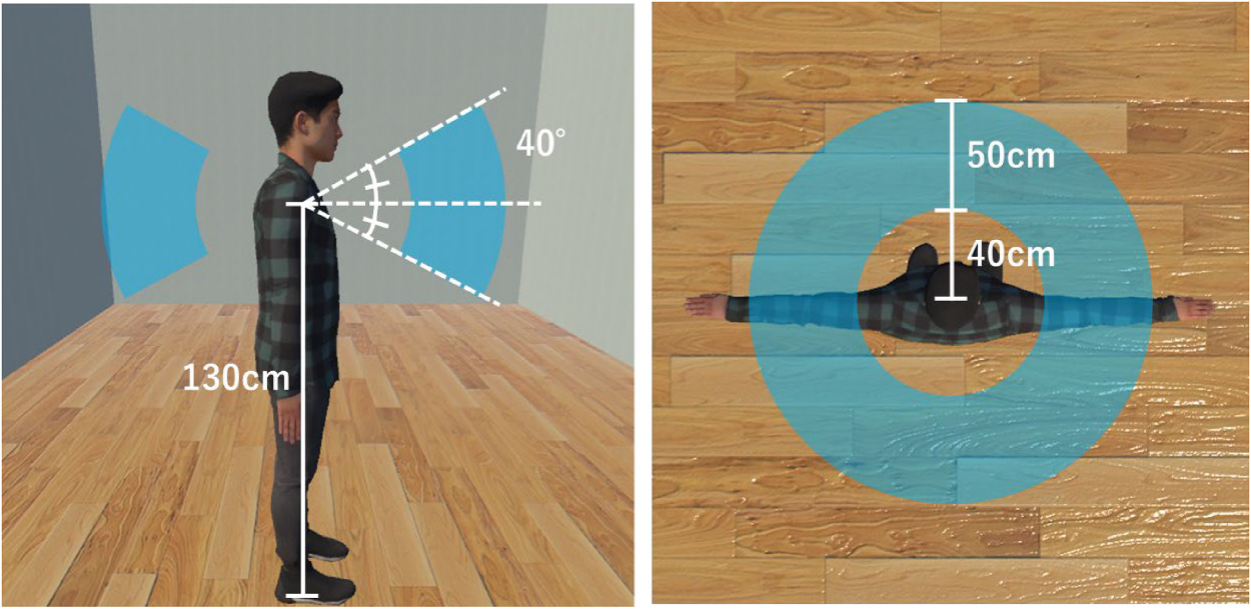
Target areas in each condition. The targets appeared at random points inside a ring-like 3-dimensional volume which was a selected section of a sphere that had its center at the center of the avatar, 130 cm above the floor level as shown in blue. The cross-section of the selected volume is shown on left (only the volume making an angle of 40° with the floor plane upwards and downwards) and included only the volume ranging from 40 to 50 cm radius as shown on right.

Participants controlled a male avatar of height 175 cm in a virtual room to perform a reaching task that involved reaching spherical targets of 15 cm diameter. The targets appeared at random positions inside the 3D space shown in Figure 3. Since the targets appeared randomly around the avatar including the back, we placed a mirror of height 150 cm and width 200 cm, 200 cm in front of the avatar. The mirror was placed to guide the participants to the targets while also allowing them to see a full body reflection of their avatar. The participants were instructed to watch the target directly while reaching instead of only looking through the mirror. The vibration motor on the corresponding palm vibrated for 3 ms on each successful target reach and the target erased. The next target appeared 3 s later. This repeated for 5 min in each session. There were natural and unnatural sessions as well as synchronous and asynchronous sessions for each avatar type. In the natural avatar sessions, the movements of the participants read from motion captures in real time were reflected onto the avatar without any alterations. In the unnatural avatar sessions, the lower arms of the virtual avatar were programmed to bend in reverse directions. In the synchronous sessions, the timing of the movements of the virtual avatar matched the timing of the movements of the participants. In the asynchronous sessions, the movements of the virtual avatar were delayed for a second compared to the movements of the participant. At the end of each reaching task session, a knife appeared in the virtual environment and penetrated the lower arm of the avatar as shown in Figure 2. HMD was blacked out 10 s after the knife appeared and the session ended.

### Procedure

The experiment consisted of 8 sessions (2 avatar types × 2 synchronicity conditions × 2 repetitions). Each participant performed the experiment on two separate days where they controlled either the natural avatar, or the unnatural avatar only on each day. Half of the participants controlled the natural avatar on their first day while the other half controlled the unnatural avatar on their first day. Synchronous and asynchronous sessions were performed in a randomized order. Participants wore motion capture suits, the HMD, the vibration motors on their palms, and transmitter to measure skin conductance on their wrists (with cables attached to electrodes on their fingers) and performed the reaching task while standing in each session. SCR was calculated as the difference between the maximum value detected in a 5 s post-stimulus (knife) time window and the baseline calculated as the average value of a 2 s pre-stimulus time window (in each session). At the end of each session, they answered an embodiment questionnaire. The order of the questions was randomized each time for each participant. Answers were accepted in a 7-point Likert scale from −3 to +3 where −3 meant “Strongly disagree” and +3 meant “Strongly agree”. Questionnaire items were created referring to previous work (Gonzalez-Franco & Peck, 2018) and are shown in Table 1. Senses of body ownership and agency were calculated as follows.

**Table 1.** Questionnaire Items.

Q1	I felt as if the virtual body I saw was my own body
Q2	I felt as if the virtual body I saw was someone else
Q3	I felt as if the virtual body I saw in the mirror was my own body
Q4	I felt as if the virtual body I saw in the mirror was someone else
Q5	I felt like I could control the virtual body as if it were my body
Q6	I felt like the virtual body was moving by itself

Sense of body ownership = (Q1−Q2) + (Q3−Q4)

Sense of agency = Q5−Q6

## Results

Senses of body ownership and agency for both avatars were higher in the synchronous condition compared to the asynchronous condition.

**Figure 4.**
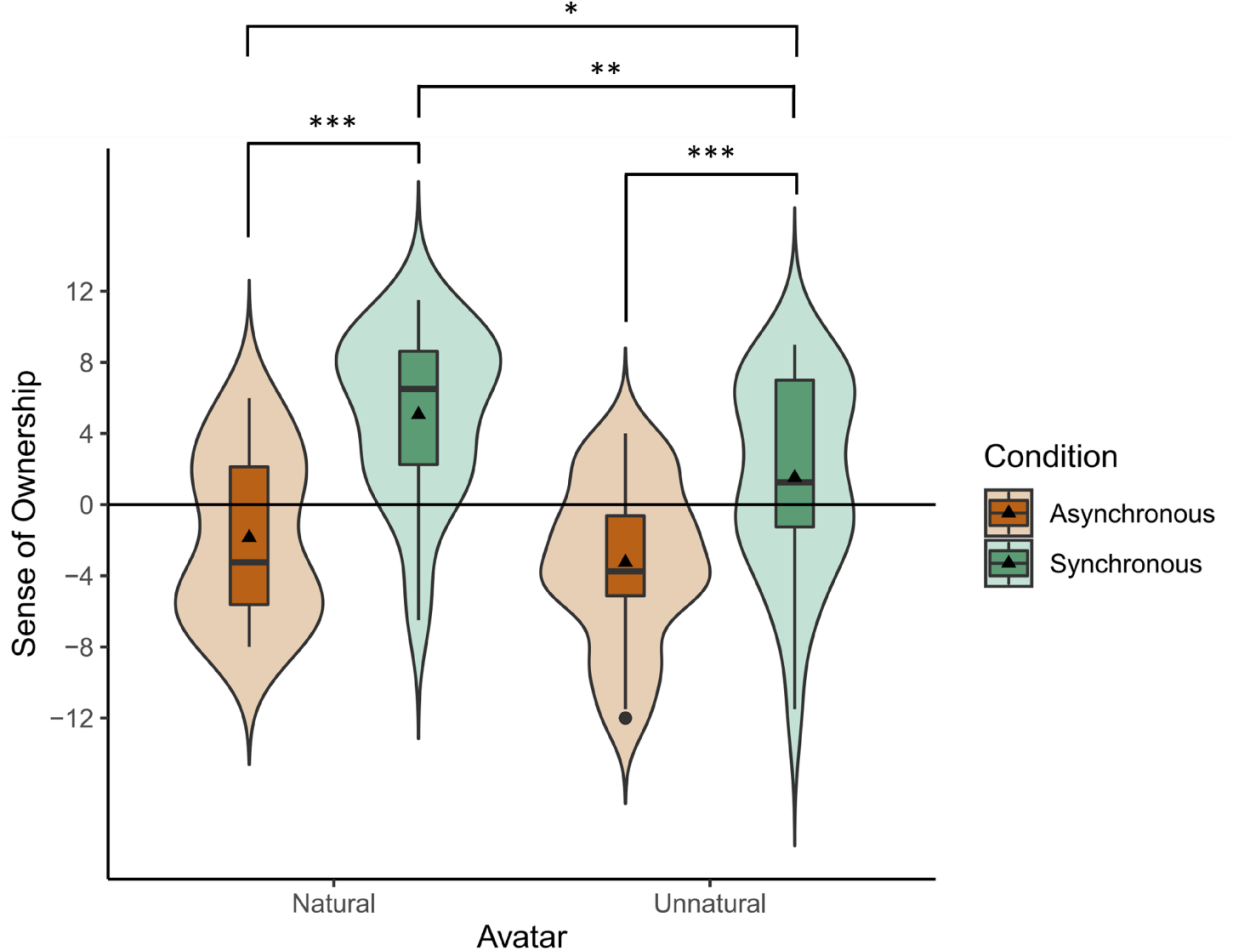
Sense of body ownership towards natural and unnatural avatars in asynchronous and synchronous conditions. Each box indicates the interquartile range (IQR), the range between 25th and 75th percentiles. The horizontal lines and the solid triangles in the boxes indicate medians and means respectively. The whiskers extend from the hinge to the largest and smallest values no further than 1.5 * IQR from the hinge. Outliers are shown in black dots. Surrounding violin plots depict the kernel density distributions. The ownership towards both avatars were significantly higher in the synchronous condition than in the asynchronous condition. In the synchronous condition, the ownership towards unnatural avatar was lower than that towards the natural avatar.

**Figure 5.**
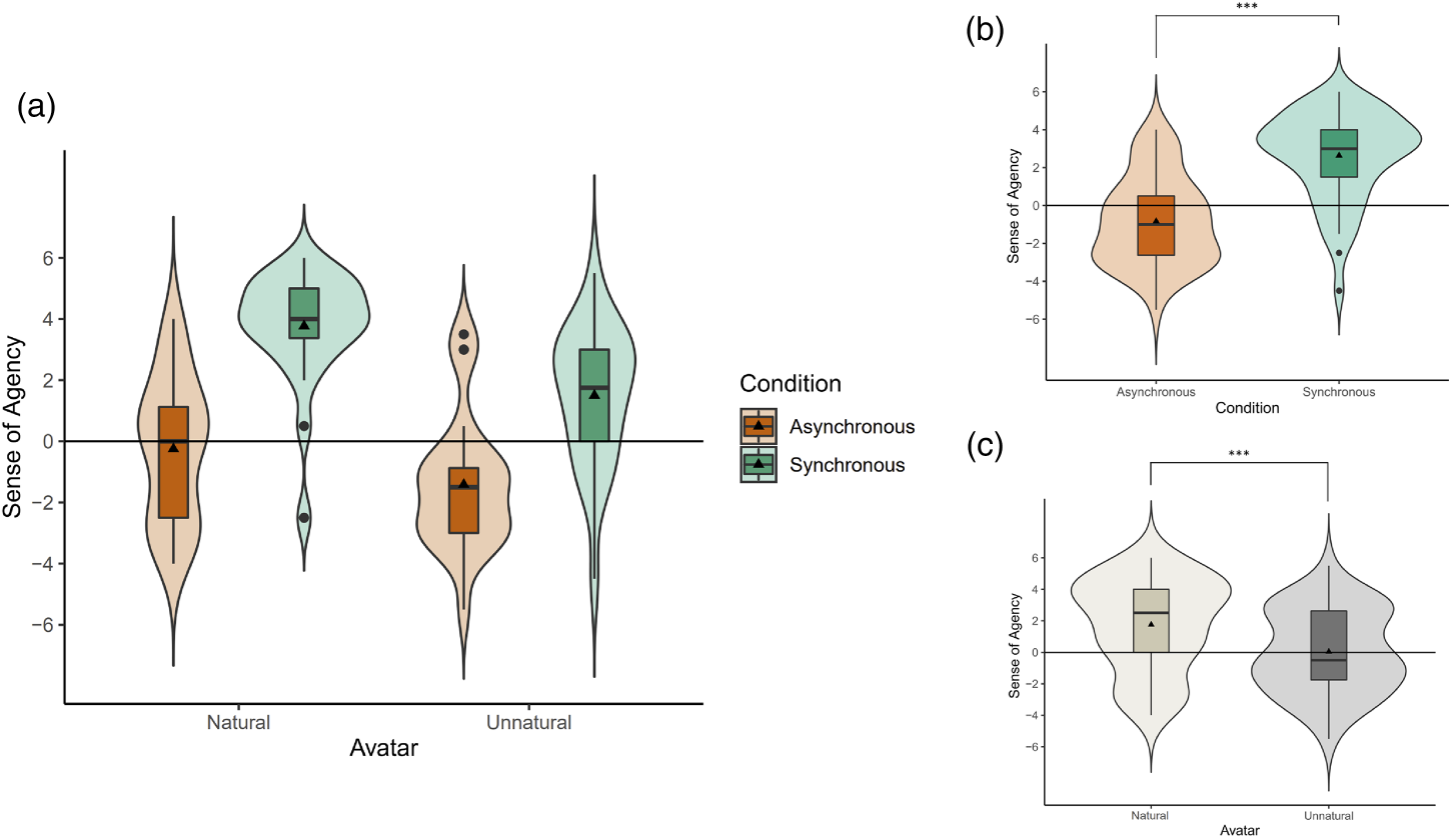
Sense of agency towards natural and unnatural avatars in asynchronous and synchronous conditions. Each box indicates the interquartile range (IQR), the range between 25th and 75th percentiles. The horizontal lines and the solid triangles in the boxes indicate medians and means respectively. The whiskers extend from the hinge to the largest and smallest values no further than 1.5 * IQR from the hinge. Outliers are shown in black dots. Surrounding violin plots depict the kernel density distributions. (a) Sense of agency of each avatar in each condition. (b) Main effect on synchronicity: the overall sense of agency in the synchronous condition was higher than that in the asynchronous condition. (c) Main effect on avatar type: the overall sense of agency towards the natural avatar was higher than that towards the unnatural avatar.

Figures 4 and 5 summarize the results of the questionnaires. For the questionnaire scores of both body ownership and agency, we first tested if the data violated normality, and then applied non-parametric 2-way ANOVA (2 natural/unnatural × 2 synchronous/asynchronous) if the data deviated from normality. These procedures of analysis were determined before conducting the experiment. The ownership questionnaire (Q1 to Q4) had a Cronbach's alpha of 0.879 showing a good internal consistency. Negative items Q2, and Q4 of the questionnaire were reversed for the calculation of Cronbach's alpha.

First, we calculated the sense of body ownership (Figure 4) towards the two avatars (natural and unnatural) in the two synchronicity conditions (synchronous and asynchronous). A two-way repeated measures ANOVA was conducted since the data were normally distributed according to the results of Shapiro–Wilk tests (asynchronous condition of natural avatar: W = .920, *p* = .058, synchronous condition of natural avatar: W = .929, *p* = .095, asynchronous condition of unnatural avatar: W = .959, *p* = .426, synchronous condition of unnatural avatar: W = .930, *p* = .099). There was a significant main effect on the avatar type (F(1,23) = 8.876, *p* = .007, $\eta_{p}^{2}$ =.279), a significant main effect on synchronicity (F(1,23) = 49.872, *p* < .001, $\eta_{p}^{2}$ =.684), and a significant interaction between the avatar type and synchronicity (F(1,23) = 5.833, *p* = .024, $\eta_{p}^{2}$ =.202), and three significant simple main effects were observed. First, the ownership towards the natural avatar was significantly higher in the synchronous condition than in the asynchronous condition (F(1,23) = 55.208, *p* < .001, $\eta_{p}^{2}$ =.706). Next, the ownership towards the unnatural avatar was also significantly higher in the synchronous condition than in the asynchronous condition (F(1,23) = 25.357, *p* < .001, $\eta_{p}^{2}$ =.524). Lastly, in the synchronous condition, the ownership towards the natural avatar was significantly higher than that towards the unnatural avatar (F(1,23) = 12.465, *p* = .002, $\eta_{p}^{2}$ =.352). In the asynchronous conditions, there was no significant difference in ownership between the natural and unnatural avatars (F(1,23) = 2.558, *p* = .123, $\eta_{p}^{2}$ =.100). Furthermore, Holm's post-hoc comparisons revealed that the ownership towards the unnatural avatar in the synchronous condition was significantly higher than the ownership towards the natural avatar in the asynchronous condition (*t*(22) = 2.651, *p* = .029).

Next, we calculated the sense of agency (Figure 5) towards the two avatars (natural and unnatural) in the two synchronicity conditions (synchronous and asynchronous). The sense of agency questionnaire (Q5 to Q6) had a Cronbach's alpha of 0.832 showing a good internal consistency. Negative item Q6 of the questionnaire was reversed for the calculation of Cronbach's alpha.

A two-way repeated measures ANOVA with ART (aligned rank transformation) procedure (Wobbrock et al., 2011) was conducted since some of the data significantly deviated from normality according to the results of Shapiro–Wilk tests (asynchronous condition of natural avatar: W = .943, *p* = .189, synchronous condition of natural avatar: W = .827, *p* < .001, asynchronous condition of unnatural avatar: W = .907, *p* = .030, synchronous condition of unnatural avatar: W = .951, *p* = .281). There was no significant interaction between the avatar type and synchronicity (F(1,23) = 3.420, *p* = .077). However, there were significant main effects for both the avatar type, and synchronicity with the sense of agency towards the natural avatar being significantly higher than that towards the unnatural avatar (F(1,23) = 21.031, *p* < .001, $\eta_{p}^{2}$ =.478) for both synchronicity conditions, and the sense of agency in the synchronous condition being significantly higher than the sense of agency in the asynchronous condition (F(1,23) = 99.985, *p* < .001, $\eta_{p}^{2}$ =.813) for both avatars.

### Skin Conductance Response Did Not Show Significant Differences Among Avatar Type or Synchronicity

**Figure 6.**
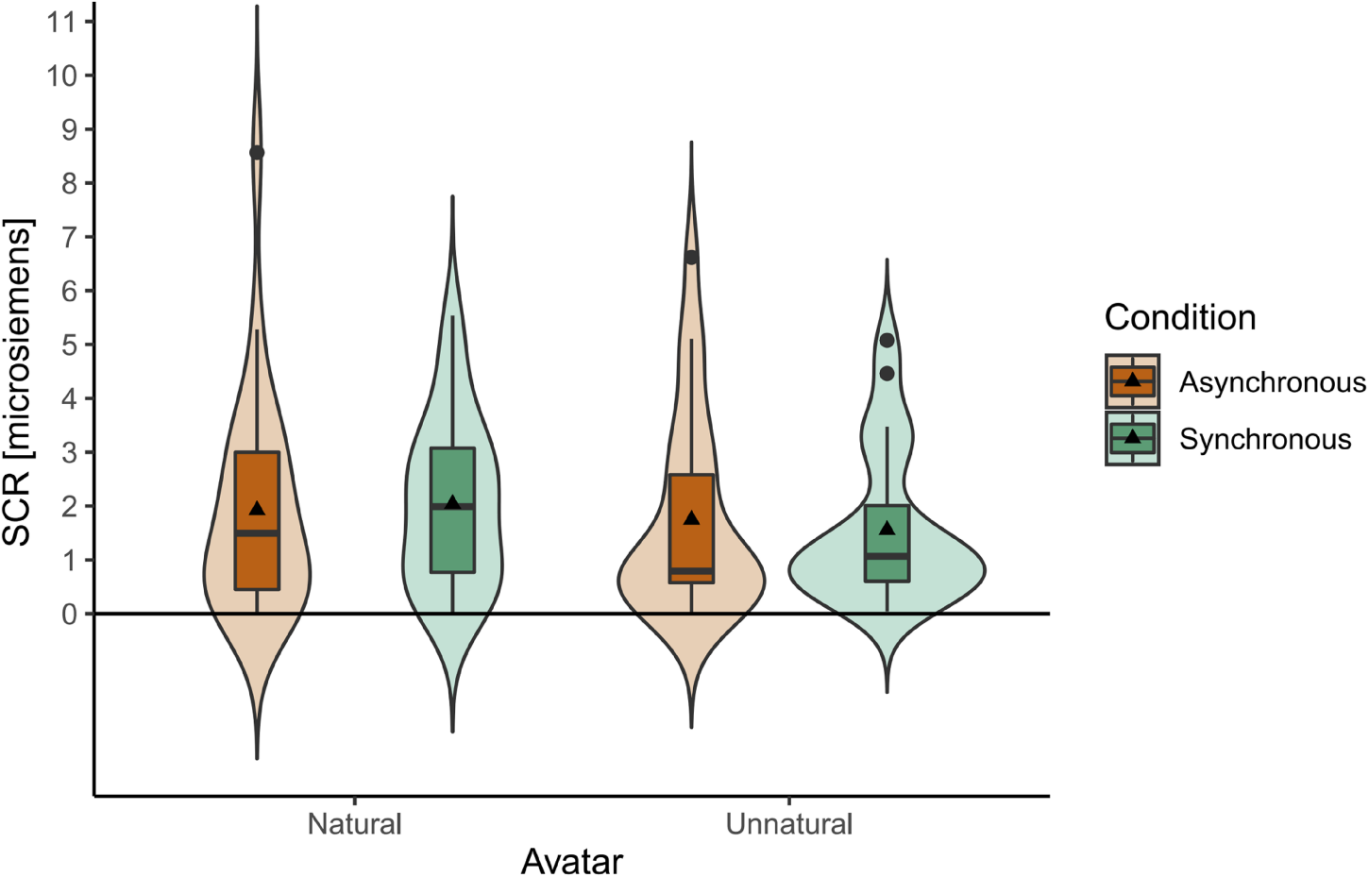
Skin conductance response of participants when the knife stabbed the arm of the natural and unnatural avatars in asynchronous and synchronous conditions. Each box indicates the interquartile range (IQR), the range between 25th and 75th percentiles. The horizontal lines and the solid triangles in the boxes indicate medians and means respectively. The whiskers extend from the hinge to the largest and smallest values no further than 1.5 * IQR from the hinge. Outliers are shown in black dots. Surrounding violin plots depict the kernel density distributions. No significant differences were observed between the avatars or synchronicity conditions.

Figure 6 summarizes the results of SCR. SCR of each participant was calculated when the threatening stimulus (the virtual knife) appeared, as the difference between the maximum value detected in a 5 s post-stimulus time window and the baseline calculated as the average value of a 2 s pre-stimulus time window.

A two-way repeated measures ANOVA with ART procedure (Wobbrock et al., 2011) was conducted since some of the data significantly deviated from normality according to the results of Shapiro–Wilk tests (asynchronous condition of natural avatar: W = .820, *p* < .001, synchronous condition of natural avatar: W = .945, *p* = .212, asynchronous condition of unnatural avatar: W = .819, *p* < .001, synchronous condition of unnatural avatar: W = .833, *p* = .001). There was no significant interaction between the avatar type and synchronicity (F(1,23) = 2.386, *p* = .136). There were no significant main effects either (avatar type: F(1,23) = 1.288, *p* = .268, synchronicity: F(1,23) = 3.522, *p* = .073).

One sample tests (student *t*-tests for normally distributed data, and Wilcoxon signed-rank tests for data that violated normality) showed that the skin conductance of participants significantly increased (*p* < .001) in response to the knife stimulus compared to the calculated baseline in all conditions (asynchronous condition of natural avatar: V = 300, *p* < .001, effect size = 1.000, synchronous condition of natural avatar: *t*(23) = 6.445, *p* < .001, *d* = 1.316, asynchronous condition of unnatural avatar: V = 300, *p* < .001, effect size = 1.000, synchronous condition of unnatural avatar: V = 300, *p* < .001, effect size = 1.000).

Next, we checked if there were correlations between SCR and ownership in each condition. Here, we first conducted Shapiro–Wilk tests for bivariate normality and calculated Spearman's rho for the data that violated normality assumptions and Pearson's *r* for the data that did not violate normality assumptions (asynchronous condition of natural avatar: W = .825, *p* < .001, synchronous condition of natural avatar: W = .921, *p* = .061, asynchronous condition of unnatural avatar: W = .824, *p* < .001, synchronous condition of unnatural avatar: W = .858, *p* = .003). No significant positive correlations between ownership and SCR were observed in any condition (asynchronous condition of natural avatar: rho = −.338, *p* = .947, synchronous condition for natural avatar: *r* = −.195, *p* = .820, asynchronous condition of unnatural avatar: rho = −.227, *p* = .857, synchronous condition of unnatural avatar: rho = .019, *p* = .465).

Next, we checked if there were positive correlations between SCR and sense of agency in each condition. Here, we first conducted Shapiro–Wilk tests for bivariate normality and calculated Spearman's rho for the data that violated normality assumptions and Pearson's *r* for the data that did not violate normality assumptions (asynchronous condition of natural avatar: W = .815, *p* < .001, synchronous condition of natural avatar: W = .829, *p* < .001, asynchronous condition of unnatural avatar: W = .819, *p* < .001, synchronous condition of unnatural avatar: W = .938, *p* = .145). No significant positive correlations between ownership and SCR were observed in any condition (asynchronous condition of natural avatar: rho = −.234, *p* = .864, synchronous condition for natural avatar: rho = −.083, *p* = .651, asynchronous condition of unnatural avatar: rho = .024, *p* = .455, synchronous condition of unnatural avatar: *r* = −.238, *p* = .868).

## Discussion

We experimentally investigated how temporal visuomotor synchrony affects the sense of embodiment towards an avatar of which the spatial visuomotor synchrony of lower arms was disrupted with inversed, biomechanically impossible movements. We compared the senses of body ownership and agency towards this unnatural avatar against those towards a natural avatar in the presence and absence of temporal visuomotor synchrony. The results showed that temporal visuomotor synchrony causes a significant increase in sense of body ownership towards the unnatural avatar in a similar manner shown towards a natural avatar, suggesting that body ownership illusions can be created even when movements of an avatar are spatially altered from the movements of its user in a way that is biomechanically impossible as a human, as long as the real movements temporally synchronize with the virtual movements. However, in the presence of temporal visuomotor synchrony, the sense of body ownership towards the natural avatar was higher than that towards the unnatural avatar suggesting that both spatial and temporal visuomotor synchronizations are necessary for strong inductions of body ownership. As possible reasons behind the lower sense of embodiment towards the unnatural avatar, two main factors can be thought of.

First, since sense of embodiment is related to visuoproprioceptive synchrony (Dummer et al., 2009; Kalckert & Ehrsson, 2012; Kalckert & Ehrsson, 2014; Tsakiris et al., 2006; Walsh et al., 2011) as well in addition to the visuomotor synchronizations, the multi-sensory conflict arising from the felt proprioception as well as the motor feedback from the real arm not coinciding with the observed virtual arm can be pointed out as a reason.

Second, a number of previous studies point to the difficulty of humans/infants to correctly recognize bodies with inversions, physically impossible structures, or biomechanically impossible body movements (Inoue & Kitazaki, 2010; Morita et al., 2012; Petit & Harris, 2005; Reed et al., 2003). Our sense of body ownership may depend on recognizing the stimuli as well as understanding the coherence of the current sensory input with the pre-existing cognitive representations of the body (Tsakiris & Haggard, 2005). In the case of the unnatural avatar, biomechanically impossible movements conflict with the body recognition as well as pre-existing cognitive representation we have for human arms making it a possible reason behind the lower embodiment despite the temporal synchrony. However, further studies are required to conclude whether body recognition is correlated to embodiment of first-person avatars, and also whether long-term training with such biomechanically impossible movements can lead to higher embodiment.

Another interesting observation we made in this study is the fact that the sense of body ownership towards the unnatural avatar with temporal synchrony was significantly higher compared to that towards the natural avatar without temporal synchrony, suggesting that temporal visuomotor synchrony in the absence of spatial visuomotor synchrony results in higher sense of body ownership compared to having spatial synchrony in the absence of temporal visuomotor synchrony. Therefore, while we conclude that spatiotemporal visuomotor synchronizations are necessary to induce stronger body ownership illusions, we also highlight the superiority of temporal visuomotor synchrony compared to spatial visuomotor synchrony when it comes to body ownership.

As a physiological measurement of body ownership, we also recorded the skin conductance of participants. They did respond to the threatening stimulus to the lower arm with a significant arousal that caused an increase in their skin conductance levels. However, the increase in skin conductance showed no significant differences between the experiment conditions suggesting that the knife moving towards the lower arm of the avatar itself was perceived threatening regardless of the synchronicity or the avatar type. A reason for this could be the fact that the knife appeared in front of the participants and moved towards them possibly threatening the self-location of the real body of the participants in all conditions. We observed no correlations between skin conductance and body ownership or between skin conductance and sense of agency. While changes in skin conductance may be a good measure of body ownership towards objects like separate rubber hands (Armel & Ramachandran, 2003; Tsuji et al., 2013), and partner-controlled limbs (Hapuarachchi & Kitazaki, 2022; Wegner et al., 2004), it may not be very reliable for measuring full body illusions according to our results due to lack of evidence supporting the subjective ratings for body ownership. Therefore, other measurements such as proprioceptive drifts (Botvinick & Cohen, 1998; Costantini & Haggard, 2007; Tsakiris & Haggard, 2005; Tosi et al., 2023) and changes in body schema and representation (Bertoni et al., 2023; Hapuarachchi et al., 2023; Matamala-Gomez et al., 2020) following previous work may better suit as alternative objective measurements for body ownership in future experiments.

Sense of agency did not show a significant interaction between the avatar type and synchronicity. However, the overall sense of agency for both avatars was higher with visuomotor synchrony, compared to having a time delay between visual and real movements. This suggests that temporal visuomotor synchrony significantly enhances the sense of agency for both natural and unnatural avatars meaning that even when the movements are spatially mismatched, temporal synchrony between virtual and real movements plays an important role in making participants feel like they are in control of the movements of their avatars. Previous studies have shown that in cases of discrepancies, the altered sensory feedbacks might nevertheless be accepted as the sensory outcome of one's own movement and can be used for the essential continuous recalibration of one's action predictions and motor behavior (Synofzik et al., 2006). Our results expand this understanding of sense of agency by adding the necessity of temporal synchrony for enhancing such motor adaptations. However, the overall sense of agency was higher for the natural avatar compared to that for the unnatural avatar regardless of temporal visuomotor synchrony. These results suggest that while inversing lower arm movements do cause a significant drop in sense of agency compared to having natural movements, presence of visuomotor synchrony improves sense of agency for both types of avatars.

Overall, while previous studies have shown that synchronous visuomotor stimulations can induce a sense of ownership over virtual arms or bodies, our study expands this understanding by demonstrating that temporal synchrony can enhance sense of embodiment even when the avatar's movements are biomechanically impossible and spatially mismatching. Our results also point out that for body ownership illusions, temporal synchronies may be superior to spatial synchronies. However, to gain a deeper understanding on visuomotor adaptations with and without temporal and spatial synchronies, further experiments have to be conducted in the future that also monitor learning and changes in perceived body ownership and agency over time.

Finally, we would like to suggest that utilizing technologies such as VR, wearable robotics, and autonomous prosthetic limbs can allow us to extend our range of motion beyond our biological bodies. Especially when it comes to prosthetics, usually amputees tend to replace lost body parts with prostheses that move with the same human biological constraints. However, if we can learn how to embody movements beyond such typical human movements, there may be a future where augmented bodies with biologically impossible movements would be frequently embodied by humans to get various tasks done efficiently. With such unique prostheses amputees may even be at a physical advantage over healthy individuals in the future. Therefore, we believe that more research on embodying various types of biomechanically impossible avatars, robots, prostheses etc. should be encouraged.

### Limitations of the Study

The participant population was not very diverse with regards to gender and age in this study. Gender and age can influence how individuals perceive and react to various situations and lack of diversity can introduce bias into the results (Brotto et al., 2021). Therefore, a replication of this study with a larger diversity with regards to gender, age, and demography may provide more reliable results.

We measured SCR as a countermeasure to get a physiological measure unaffected by demand characteristics of the participants associated with the questionnaire items on sense of agency and body ownership. However, from skin conductance data, as discussed previously, we did not get a result supporting the subjective ratings. Previous studies have shown that participants tend to respond in ways that confirmed the hypothesis, yet this tendency depends on attitudes toward the experiment or the experimenter and other individual differences (Nichols & Maner, 2008). Other recent studies also provide convincing evidence showing that participants can guess the aims of body illusion experiments (Lush, 2020; Lush et al., 2021; Reader, 2022). Therefore, the lack of action to minimize such demand characteristics is a limitation of the study.
